# Anti-inflammatory Lignans from the Fruits of *Acanthopanax sessiliflorus*

**DOI:** 10.3390/molecules18010041

**Published:** 2012-12-21

**Authors:** Dae-Young Lee, Kyeong-Hwa Seo, Rak-Hun Jeong, Sang-Min Lee, Geum-Soog Kim, Hyung-Jun Noh, Seung-Yu Kim, Gye-Won Kim, Ji-Young Kim, Nam-In Baek

**Affiliations:** 1 Herbal Crop Utilization Research Team, National Institute of Horticultural and Herbal Science, RDA, Eumseong 369-873, Korea; 2 Graduate School of Biotechnology and Department of Oriental Medicinal Materials & Processing, Kyung Hee University, Yongin 446-701, Korea; 3 Brewing Research Center, Han Kyung National University, Ansung 456-749, Korea

**Keywords:** *Acanthopanax sessiliflorus*, lignan, acanthosessilin A, nitric oxide

## Abstract

A new lignan, named acanthosessilin A (**1**), as well as eight known lignan and lignan glycosides **2**−**9** were isolated from an ethanolic extract of *Acanthopanax sessiliflorus* fruits. The chemical structures were determined by spectroscopic methods, including HR-EIMS, 1D NMR (^1^H, ^13^C, DEPT), 2D NMR (gCOSY, gHSQC, gHMBC, NOESY), and IR spectroscopy. All isolated compounds were tested for the ability to inhibit LPS-induced nitric oxide production in RAW264.7 macrophages.

## 1. Introduction

*Acanthopanax sessiliflorus* (Rupr. et Maxim) Seem, belonging to the Araliaceae family, is widely distributed in Korea, China, and Japan. The bark and twigs of *Acanthopanax* species are traditionally used in Korea as anti-rheumatoid arthritis, anti-inflammatory, and anti-diabetic drugs and are recognized to have ginseng-like activities [1,2]. Previous studies on its phytochemicals resulted in the isolation of lignans from the leaves and roots of *Acanthopanax* species [3,4,5], and eleutheroside E has been identified as a major compound in the fruits of *Acanthopanax* species [6]. Lignans are thought to be the major active constituents of these plants and are believed to play essential roles in the treatment of diseases [7,8]. However, most phytochemical and pharmacological studies have mainly focused on the leaves, bark, and roots of *Acanthopanax* species, and only a few reports have investigated the fruits. *Acanthopanax* species are native medicinal plants and the fruits of *Acanthopanax* species have been used as a remedy to “wipe out evil wind” in traditional medicine [9]. To further investigate the bioactive constituents derived in the fruits of these species, the present phytochemical study was initiated.

We report herein on the isolation of a new 3,4-dibenzylfuran lignan (**1**) from the fruits of *A. sessiliflorus*, together with eight known compounds **2**−**9**, and the structural determination of these substances using extensive spectroscopic methods. Several previous studies have provided evidence for the anti-inflammatory effects of extracts and components from *Acanthopanax* species [10,11,12]. Therefore, isolated compounds **1**−**9** were evaluated for anti-inflammatory activities through the measurement of nitrite, a soluble oxidation product of nitric oxide (NO), in lipopolysaccharide (LPS)-induced RAW 254.7 macrophage cells.

## 2. Results and Discussion

**Figure 1:**
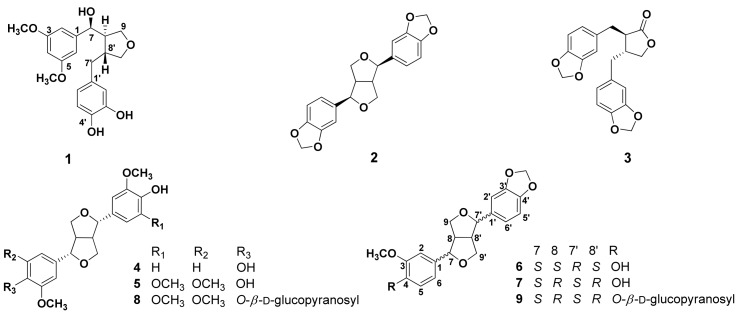
Chemical structures of isolated compounds **1**−**9**.

A 70% ethanolic extract of dried *A. sessiliflorus* fruits was suspended in H_2_O and extracted with EtOAc. The EtOAc soluble fraction was concentrated under reduced pressure to produce a residue that was subjected to multiple chromatographic steps using Sephadex LH-20, silica gel, and reversed-phase C18, yielding compounds **1**−**9** (Figure 1).

**Figure 2:**
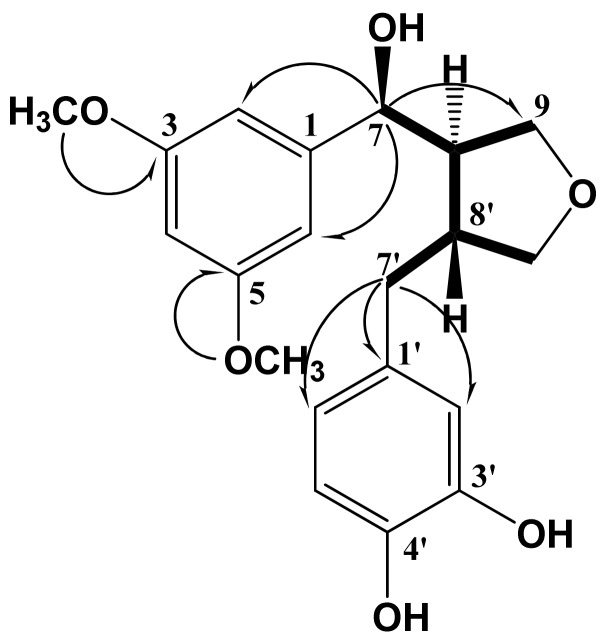
Key ^1^H−^1^H COSY (bold dash) and HMBC (arrow) correlations of compound **1**.

Compound **1**, obtained as colorless crystals from methanol, exhibited a UV absorption maximum at 282 nm. The molecular formula was determined to be C_20_H_24_O_6_ from the molecular ion peak [M]^+^ at *m/z* 360.1552 (calcd for C_20_H_24_O_6_, 360.1572) in the HR-EIMS. IR absorption bands at 3,430, 1,648, and 1512 cm^−1^ were characteristic of hydroxyl and aromatic groups. The ^1^H-NMR spectrum showed three aromatic proton signals with *J*_4_ coupling at δ_H_ 6.90 (1H, d, *J* = 3.2 Hz, H-4), 6.76 (1H, overlapped, H-2), and 6.75 (1H, overlapped, H-6), which were assigned to a 1,3,5-trisubstituted benzene moiety. Three other aromatic proton signals at δ_H_ 6.78 (1H, d, *J* = 2.0 Hz, H-2'), 6.70 (1H, d, *J* = 8.0 Hz, H-6'), and 6.63 (1H, dd, *J* = 8.0, 2.0 Hz, H-5') corresponded to another 1,2,4-trisubstituted benzene moiety. A doublet oxygenated methine proton signal at δ_H_ 4.74 (1H, *J* = 6.8 Hz), assigned to H-7, and two oxygenated methyl proton signals at δ_H_ 3.82 (3H) and 3.83 (3H) for two methoxy groups were observed. Two oxygenated methylene proton signals were observed at δ_H_ 3.97 (1H, dd, *J* = 8.4, 6.8 Hz), 3.71 (1H, dd, *J* = 8.4, 6.8 Hz), 3.82 (1H, overlapped), and 3.62 (1H, dd, *J* = 10.8, 6.4 Hz), which were assigned to H-9a, H-9b, H-9'a, and H-9'b, respectively. In the high magnetic field, two methine proton signals at δ_H_ 2.34 (1H, m, H-8) and 2.72 (1H, m, H-8'), and two methylene proton signals at δ_H_ 2.92 (1H, dd, *J* = 13.2, 4.8 Hz, H-7'a) and 2.48 (1H, dd, *J* = 13.2, 11.6 Hz, H-7'b) were observed, suggesting the presence of a furan moiety. The ^13^C-NMR spectrum showed twenty carbon signals, including two methoxy carbon signals [δ_C_ 56.3 (OMe-3,5)], confirming **1** to be a lignan. The multiplicity of each carbon was determined using a DEPT experiment. In the aromatic region, six olefin methine carbon signals [δ_C_ 122.1 (C-6'), 119.8 (C-5'), 116.1 (C-2), 115.9 (C-6), 113.3 (C-2'), and 110.6 (C-4)], two carbonated quaternary carbon signals [δ_C_ 135.7 (C-1) and 133.5 (C-1')] and four oxygenated quaternary carbon signals [δ_C_ 149.0 (C-3, 5), 147.0 (C-4'), and 145.7 (C-3')] due to the 1,3,5-tri- and 1,2,4-trisubstituted benzene moieties were observed. The oxygenated methine carbon signal at δ_C_ 84.0 (C-7) shifted downfield due to attached to heteroatom (–OH). Also, two oxygenated methylene carbon signals [δ_C_ 73.4 (C-9') and 60.4 (C-9)] and two methoxy carbon signals [δ_C_ 56.3 (3, 5-OMe)] were observed. In the high magnetic field, two methine carbon signals [δ_C_ 54.0 (C-8) and 43.8 (C-8')] and a methylene carbon signal [δ_C_ 33.6 (C-7')] were observed. With further analysis of the HSQC and DEPT 135 spectra of **1**, the proton and carbon NMR signals could be assigned (Table 1). The correlations in the ^1^H−^1^H COSY spectrum indicated key connectives of H-8 (δ_H_ 2.34) with H-7 (δ_H_ 4.74), H-8' (δ_H_ 2.72), H-9a (δ_H_ 3.97), and H-9b (δ_H_ 3.71) and H-8' (δ_H_ 2.72) with H-7'b (δ_H_ 2.48), H-9'a (δ_H_ 3.82), and H-9'b (δ_H_ 3.62) (Figure 2). In the HMBC spectrum, the long-range correlations of the two aromatic rings with the tetrahydrofuran ring were indicated by cross peaks between H-7 (δ_H_ 4.74) and C-2 (δ_C_ 116.1), C-6 (δ_C_ 115.9), and C-9 (δ_C_ 60.4) and between H-7' (δ_H_ 2.92, 2.48) and C-1' (δ_C_ 133.5), C-2' (δ_C_ 113.3), and C-6' (δ_C_ 122.1) (Figure 2). In addition, the long-range correlations between the proton signals of methoxy (δ_H_ 3.82, 3.83) and the oxygenated quaternary carbon signals of C-3, 5 (δ_c_ 149.0) were also identified. The relative stereochemistry of H-8 and H-8' **1** was identified as *trans* from the lack of NOE effect between H-8 and H-8'. The coupling constant of 6.8 Hz between H-7 and H-8, as well as the optical rotation of **1** ([α]^25^_D_ = −43.5°) suggested an *S* configuration at C-7 [13]. A lignan with 7*S* and *8R* configuration of similar structure, (*R*,4*R*)-4-[(*S*)-(hydroxy)(4-hydroxy-3-methoxyphenyl)methyl]-3-(4-hydoxy-3-methoxybenzyl)tetrahydrofuran ([α]^20^_D_ = −49°), supported the above, as reported in the literature [14]. The ^13^C-NMR spectra (C-7, C-8, C-7', C-8') and NOESY experiment of **1** was very similar to (+) tripterygiol except for the optical rotation ([α]^25^_D_ = +48.3°) and were comparable to the *epi-*THF lignan [15]. This indicates that the H-8 and H-8' are present in (7*S*,8*R*)-configuration.

Finally, the structure of **1** was determined to be 3-(3',4'-dihydroxybenzyl)-4-[(7*S*),7-hydroxy-3,5-dimethoxybenzyl]tetrahydrofuran, and named acanthosessilin A. Comparisons of NMR and MS data for the known compounds **2**–**9** with reported values led to their identification as (−)-sesamin (**2**) [16], (−)-hinokinin (**3**) [3], (+)-syringaresinol (**4**) [16], (+)-pinoresinol (**5**) [17], (+)-piperitol (**6**) [18], (+)-xanthoxylol (**7**) [19], acanthoside B (**8**) [20], and simlexoside (**9**) [21], respectively (Figure 1). Compounds **1**, **6**, **7**, and **9** were isolated from the genus *Acanthopanax* for the first time. In addition, compounds **3** and **5** were also isolated from this plant for the first time.

Previous studies have already reported on the anti-inflammatory effects of components from *A. sessiliflorus* [11,12]. Since NO is known to play an important role in the inflammatory process, inhibitors of NO production are considered as potential anti-inflammatory agents [22]. Thus, we also investigated the inhibitory effects of compounds (**1**−**9**) on NO production by using the Griess reaction to measure nitrite, a soluble oxidation product of NO, in the culture medium of LPS-induced RAW 264.7 macrophages. As shown in Table 2, compounds **3**−**7** moderately inhibited NO production with IC_50_ values of 21.56, 17.75, 10.34, 22.30, and 27.57 μM, respectively. Compounds **1**, **2**, **8**, and **9** also decreased NO production with IC_50_ values in the range of 49.94 to 65.07 μM. Some cell toxicity was observed in cells treated with compounds **3** and **9**, whereas other compounds had no influence on cell viability.

## 3. Experimental

### 3.1. General

Melting points were obtained using a Fisher-Johns Melting Point Apparatus with a microscope. Ultraviolet spectra were measured on a Shimadzu model UV-1601 spectrophotometer. CD spectra were obtained with a JASCO 715 spectropolarimeter. Optical rotations were measured on a JASCO P-1010 digital polarimeter. ^1^H-, ^13^C-, and 2D-NMR spectra were recorded on a Varian Unity Inova AS 400 FT-NMR instrument, and chemical shifts were given in δ (ppm) based on tetramethylsilane (TMS) as an internal standard. IR spectra were run on a Perkin Elmer Spectrum One FT-IR spectrometer. EIMS and HR-EIMS spectra were obtained using a JEOL JMS-700 mass spectrometer (Tokyo, Japan). Silica gel 60 (Merck, 230−400 mesh), LiChroprep RP-18 (Merck, 40−63 μm), and Sephadex LH-20 (Amersham Pharmacia Biotech., Uppsala, Sweden) were used for column chromatography (CC). Pre-coated silica gel plates (Merck, Kieselgel 60 F_254_, 0.25 mm) and pre-coated RP-18 F_254s_ plates (Merck) were used for analytical thin-layer chromatography analyses. Spots were visualized by spraying with 10% aqueous H_2_SO_4_ solution followed by heating.

### 3.2. Plant Material

The fruits of *A. sessiliflorus* were provided by the Jeongseon Agricultural Extension Center, Jeongseon, Korea in August 2009 and were identified by Prof. Dae-Keun Kim, College of Pharmacy, Woo Suk University, Jeonju, Korea. A voucher specimen (KHU090809) was reserved at the Laboratory of Natural Products Chemistry, Kyung Hee University, Yongin, Korea.

### 3.3. Extraction and Isolation

The air-dried fruits of *A. sessiliflorus* (10 kg) were powdered and extracted three times with 36 L of aqueous 70% EtOH at room temperature for 24 h. After concentration *in vacuo*, the EtOH extract (2,012 g) was suspended in H_2_O (3 L) and then partitioned with EtOAc (3 L × 3) followed by concentration to give the EtOAc fraction (E, 118 g). Fraction E (100 g) was subjected to a silica gel CC (15 × 21 cm) using a gradient of CH_3_Cl_3_−MeOH (15:1 → 10:1 → 5:1 → 3:1 → 1:1, 2.8 L each) to yield 14 fractions (E1 to Ε14). Fraction E1 [4.3 g, elution volume/total volume (Ve/Vt) 0.01–0.07] was subjected to the silica gel CC [5 × 10 cm, *n*-hexane−EtOAc (6:1, 4.5 L)] to give compound **2** [486 mg, Ve/Vt 0.43–0.60, (silica F_254_) R_f_ 0.55, *n*-hexane−EtOAc (2:1)]. Subfraction E1-17 (500 mg, Ve/Vt 0.48–0.77) was separated by CC [RP-18 (3.5 × 4 cm), acetone−H_2_O (2:1, 1.5 L)] to give compound **3** [22.7 mg, Ve/Vt 0.40–0.45, TLC (RP-18 F_254s_) R_f_ 0.55, acetone−H_2_O (1:1)]. Fraction E3 [36.3 g, Ve/Vt 0.15–0.33] was subjected to the silica gel CC [6 × 16 cm, CHCl_3_−EtOAc (7:1, 5.5 L)] to give five subfractions (E3-1 to E3-5). CC [silica gel (3.5 × 16 cm), *n*-hexane−EtOAc (1:1, 3 L)] of subfraction E3-3 (1.80 g, Ve/Vt 0.34–0.53) gave 19 subfractions (E3-3-1 to E3-3-19). Subfraction E3-3-13 (80 mg, Ve/Vt 0.36–0.51) was separated by CC [RP-18 (3.5 × 5.5 cm), acetone−MeOH−H_2_O (1:1:3, 1.5 L)] to give compound **1** [11 mg, Ve/Vt 0.22–0.33, TLC (RP-18 F_254s_) R_f_ 0.55, acetone−MeOH−H_2_O (1:2:1)]. Fraction E8 (8.98 g, Ve/Vt 0.59–0.67) was fractionated using silica gel CC [4 × 12 cm, CHCl_3_−MeOH−H_2_O (16:3:1 → 13:3:1, each 3.7 L)] and yielded nine subfractions (E8-1 to E8-9). Subfraction E8-4 (1.85 g, Ve/Vt 0.45–0.58) was purified using CC [RP-18 (3.5 × 6.5 cm), MeOH−H_2_O (3:1, 1.2 L)] to give compound **4** [55 mg, Ve/Vt 0.56–0.70, TLC (RP-18 F_254s_) R_f_ 0.40, MeOH−H_2_O (5:1)]. Subfraction E8-5 (1.22 g, Ve/Vt 0.59–0.68) was fractionated using a Sephadex LH 20 CC [3 × 50 cm, MeOH−H_2_O (4:1, 1.8 L)] and yielded five subfractions (E8-5-1 to E8-5-5). Purification of subfraction E8-5-4 (222 mg, Ve/Vt 0.75–0.88) using CC [RP-18 (3 × 10 cm), EtOH−H_2_O (1:3, 0.5 L)] yielded compound **8** [44 mg, Ve/Vt 0.33–0.50, TLC (RP-18 F_254s_) R_f_ 0.70, EtOH−H_2_O (1:1)]. Subfraction E8-5-5 (146 mg, Ve/Vt 0.89–1.00) was separated by CC [RP-18 (30 × 10 cm), MeOH−H_2_O (3:1, 1 L)] to give compound **9** [20 mg, Ve/Vt 0.49–0.60, TLC (RP-18 F_254s_) R_f_ 0.50, MeOH−H_2_O (5:1)]. Fraction E9 (5.80 g, Ve/Vt 0.68–0.72) was fractionated using silica gel CC [5 × 18 cm, CH_3_Cl_3_−EtOH−H_2_O (16:3:1 → 13:3:1 → 10:3:1, each 3.2 L)] and yielded four subfractions (E9-1 to E9-4). Subfraction E9-4 (2.45 g, Ve/Vt 0.75–1.00) was chromatographed over RP-18 (5 × 5.5 cm) and eluted with MeOH−H_2_O (1:1 → 3:1, each 1.8 L) to give twenty subfractions (E9-4-1 to E-9-4-20). Subfraction E9-4-1 (282 mg, Ve/Vt 0.01–0.12) was purified over silica gel CC (4 × 12 cm) and eluted with CHCl_3_−MeOH−H_2_O (14:3:1, 2 L) to give compound **10** [40 mg, Ve/Vt 0.22–0.32, TLC (silica F_254_) R_f_ 0.65, CHCl_3_−MeOH−H_2_O (14:3:1)]. Subfraction E9-4-6 (190 mg, Ve/Vt 0.55–0.64) was separated by CC [RP-18 (30 × 10 cm), acetone−H_2_O (1:1, 1.5 L)] to give compound **6** [20 mg, Ve/Vt 0.49–0.60, TLC (RP-18 F_254s_) R_f_ 0.55, acetone−H_2_O (2:1)] and compound **7** [11 mg, Ve/Vt 0.66–0.71, TLC (RP-18 F_254s_) R_f_ 0.50, acetone−H_2_O (2:1)].

### 3.4. Spectroscopic Data

*Acanthosessilin A* (**1**). Colorless crystals, m.p.: 123−125 °C; [α]^25^_D_ −43.5° (*c* = 0.5, MeOH); CD (*c* = 2.50 × 10^−3^ M, MeOH) λ_max_ nm (*∆ε*): −0.42 (217), −0.49 (236); UV λ_max_ (MeOH) nm: 280; IR (CaF_2_ window) cm^−^^1^: 3430, 1648, 1512, 1245; EIMS *m*/*z*: 360 [M]^+^; HR-EIMS *m*/*z*: 360.1552 [M]^+^ (calcd for C_20_H_24_O_6_, 360.1572); ^1^H- and ^13^C-NMR data, see Table 1.

### 3.5. Measurement of NO Production and Cell Viability

Assays for NO production and cell viability were carried out as previously described [23]. Briefly, RAW 264.7 macrophages were harvested and seeded in 96-well plates (1 × 10^4^ cells/well) for measurement of NO production. The plates were pretreated with various concentrations of samples for 30 min and incubated with LPS (1 μg/mL) for 24 h. The amount of NO was determined by the nitrite concentration in cultured RAW264.7 macrophage supernatants using the Griess reagent. The cell viability was evaluated by MTT reduction.

## 4. Conclusions

The new compound 3-(3',4'-dihydroxybenzyl)-4-[(7*S*),7-hydroxy-3,5-dimethoxybenzyl]tetrahydrofuran, named acanthosessilin A (1), was isolated from *Acanthopanax sessiliflorus*, together with eight known lignans. According to previous investigations on *Acanthopanax* species, we have evaluated the inhibitory activities of all compounds against LPS-induced NO production in RAW264.7 macrophages. All compounds moderately inhibited NO production with IC_50_ values in the range of 10.34 to 65.07 μM. The results provide a potential explanation for the use of this plant as a herbal medicine in the treatment of inflammatory diseases, and they may be potentially useful in developing new anti-inflammatory agents.

## Figures and Tables

**Table 1:** ^1^H- (400 MHz) and ^13^C-NMR (100 MHz) data of compound **1** (in CD_3_OD, *δ* in ppm, *J* in Hz) ^a^.

No.	δ_H_	δ_C_		No.	δ_H_	δ_C_
1		135.7		1'		133.5
2	6.76 (1H, overlapped)	116.1		2'	6.78 (1H, d, *J* = 2.0 Hz)	113.3
3		149.0		3'		145.7
4	6.90 (1H, d, *J* = 3.2 Hz)	110.6		4'		147.0
5		149.0		5'	6.70 (1H, d, *J* = 8.0 Hz)	119.8
6	6.75 (1H, overlapped)	115.9		6'	6.63 (1H, dd, *J* = 8.0, 2.0 Hz)	122.1
7	4.74 (1H, d, *J* = 6.8)	84.0		7'	2.92 (1H, dd, *J* = 13.2, 4.8 Hz, H-7'a)2.48 (1H, dd, *J* = 13.2, 11.6 Hz, H-7'b)	33.6
8	2.34 (1H, *m*)	54.0		8'	2.72 (1H, *m*)	43.8
9	3.97 (1H, dd, *J* = 8.4, 6.8, H-9a)3.71 (1H, dd, *J* = 8.4, 6.8, H-9b)	60.4		9'	3.82 (1H, overlapped, H-9'a)3.62 (1H, dd, 10.8, 6.4 Hz, H-9'b)	73.4
3-OCH_3_	3.83 (3H, s)	56.3				
5-OCH_3_	3.82 (3H, s)	56.3				

^a^ Assignments were confirmed by DEPT, ^1^H−^1^H COSY, HSQC, and HMBC.

**Table 2:** Inhibitory effects of compounds **1**−**9** against LPS-Induced NO production in RAW 264.7 macrophage cells.

Compound	IC_50_ (μM) ^a^	Cell viability (%) ^b^
**1**	49.94 ± 6.56	84.81 ± 2.71
**2**	38.92 ± 2.86	95.52 ± 2.01
**3**	21.56 ± 1.19	50.21 ± 1.55
**4**	17.75 ± 1.15	80.21 ± 1.11
**5**	10.34 ± 2.37	81.50 ± 3.32
**6**	22.30 ± 1.10	84.11 ± 2.46
**7**	21.57 ± 1.28	88.43 ± 3.71
**8**	65.07 ± 8.02	82.42 ± 1.27
**9**	53.00 ± 2.75	54.52 ± 2.21
**Aminoguanidine ^c^**	6.51 ± 1.15	84.61 ± 2.50

^a^ IC_50_ value of each compound was defined as the concentration (μM) that caused 50% inhibition of NO production in LPS-activated RAW 264.7 macrophage cells. Cells were pretreated for 1 h with compounds before stimulation with LPS (1 μg/mL) for 24 h; ^b^ Cell viability indicates mean maximum inhibitory effect, at a concentration of 100 μM, expressed as a percentage inhibition of nitrite production induced by LPS (1 μg/mL) in the presence of vehicle; ^c^ Positive control. The results are averages of three independent experiments, and the data are expressed as mean ± SD.

## Supplementary Materials

^1^H-NMR, ^13^C-NMR, EIMS, and HR-EIMS spectra of **1** are available as supporting data, which can be accessed at: http://www.mdpi.com/1420-3049/18/1/41/s1.
