# Supplementary material for: Anti-inflammatory Lignans from the Fruits of Acanthopanax sessiliflorus
Source: Molecules. 2012 Dec 21;18(1):41–9. doi: 10.3390/molecules18010041 (PMC6270163; doi:10.3390/molecules18010041)

Supplementary Material

In this "Supplementary Mateiral" file for the manuscript "Anti-Inflammatory Lignans from the Fruits of *Acanthopanax sessiliflorus*", ^{1}H-NMR, ^{13}C-NMR, and EIMS, HR-EIMS spectra of new compound 1 are available here as listed below.

Contents: Pages S1–S3 ^{1}H and ^{13}C-NMR, EIMS, and HR-EIMS of **1**.

Figure S1. ^{1}H-NMR of compound **1**.

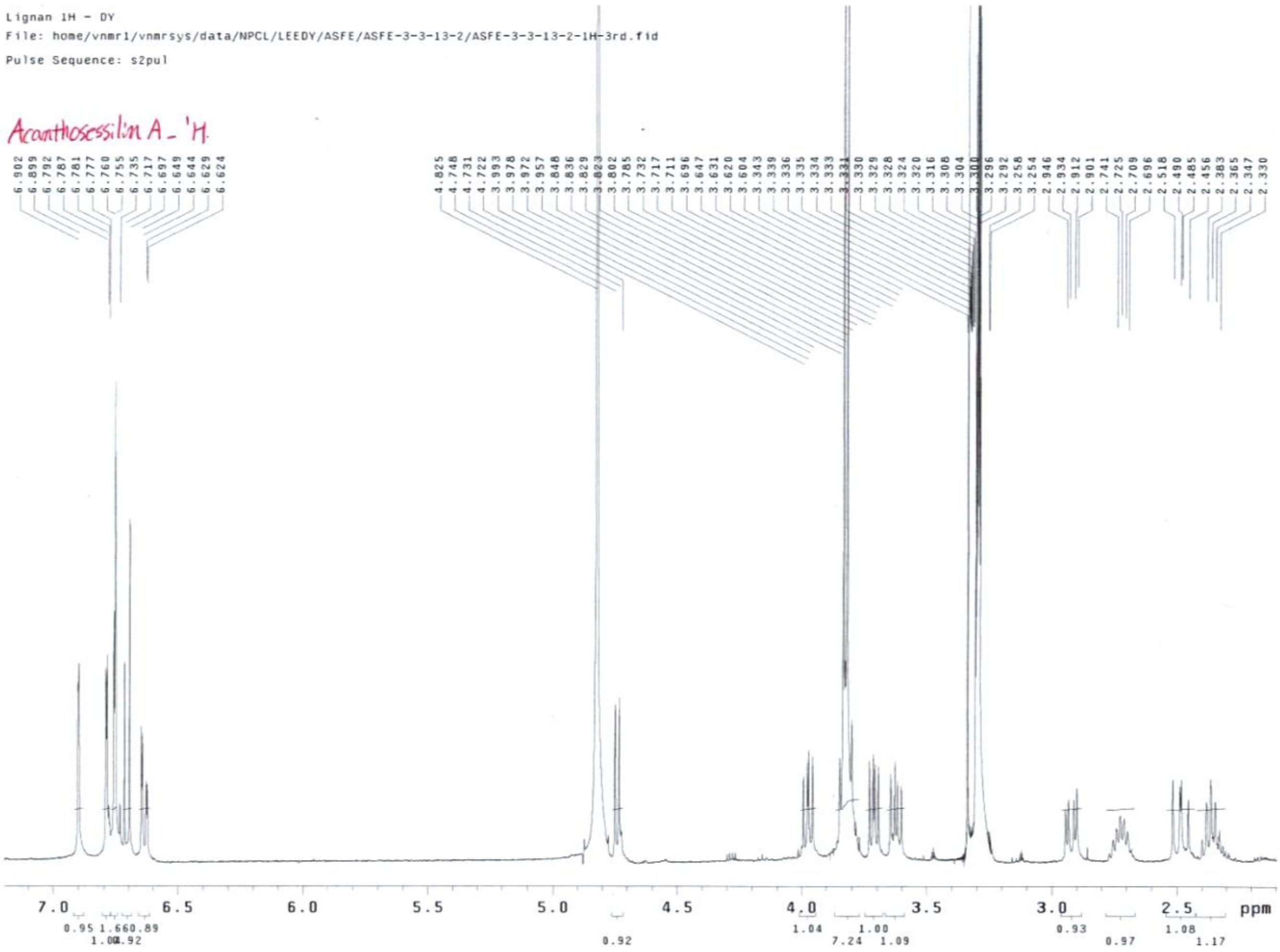

Figure S2. ^{13}C-NMR of compound **1**.

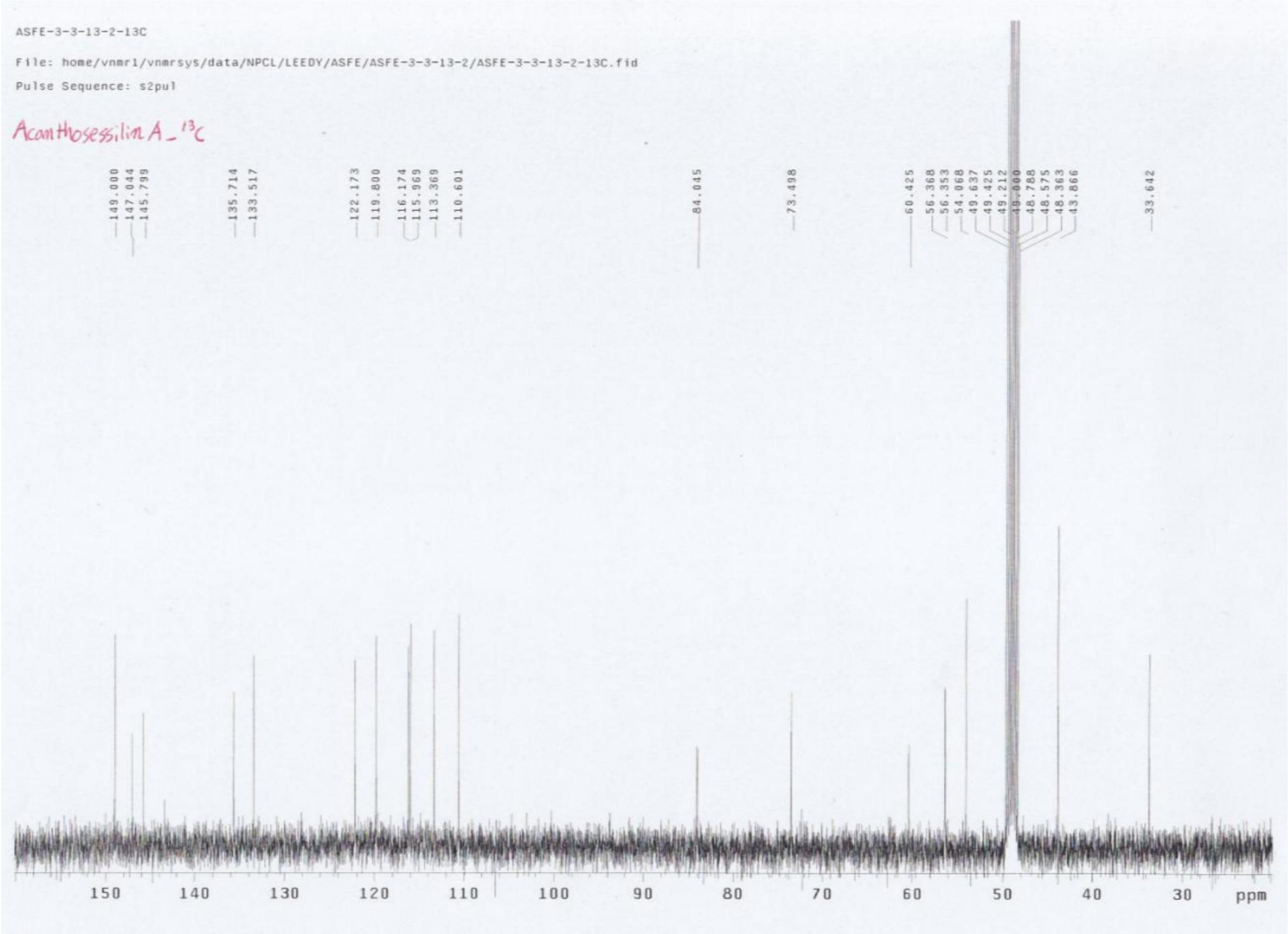

Figure S3. EIMS of compound **1**.

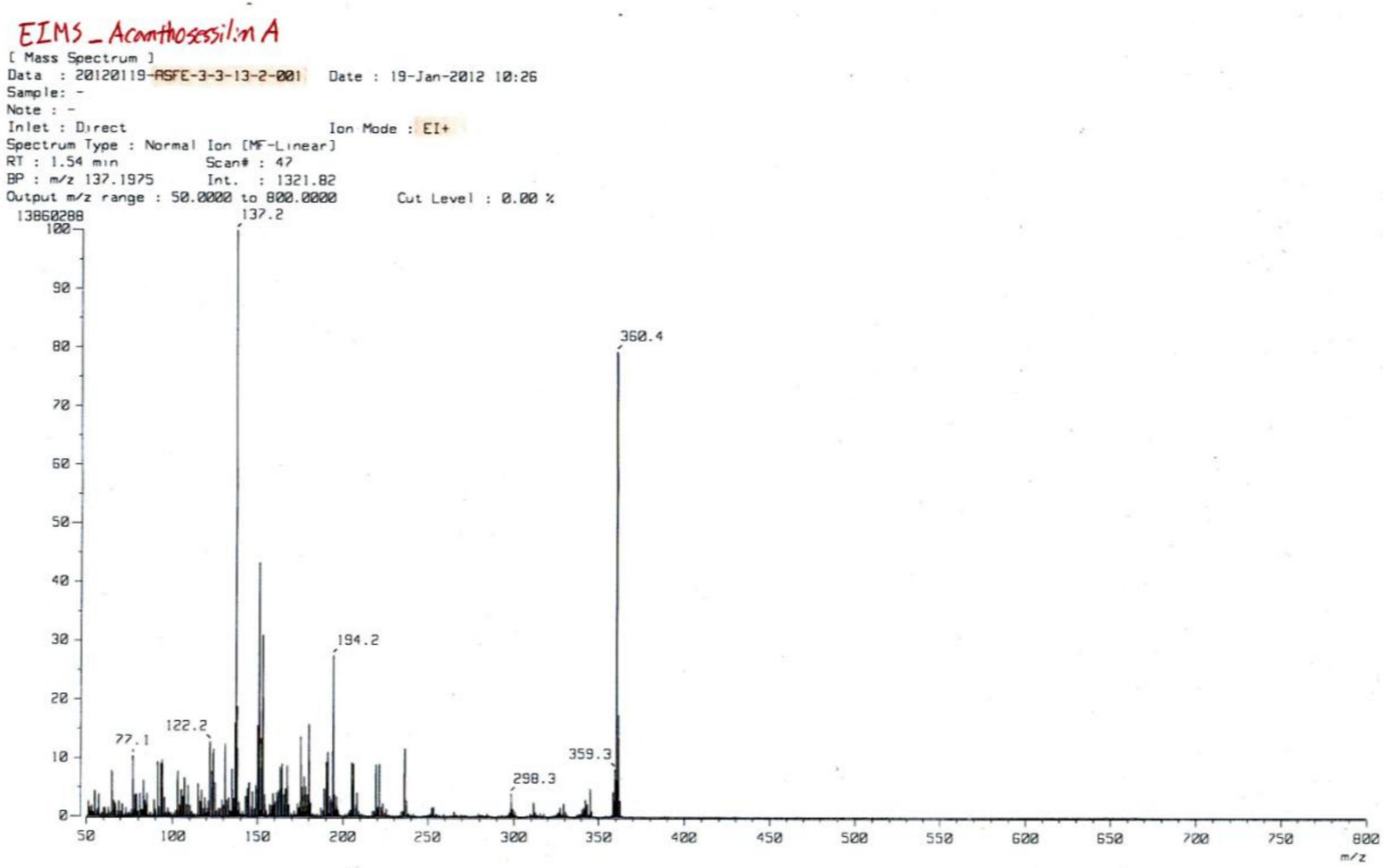

Figure S4. HR-EIMS of compound **1**.

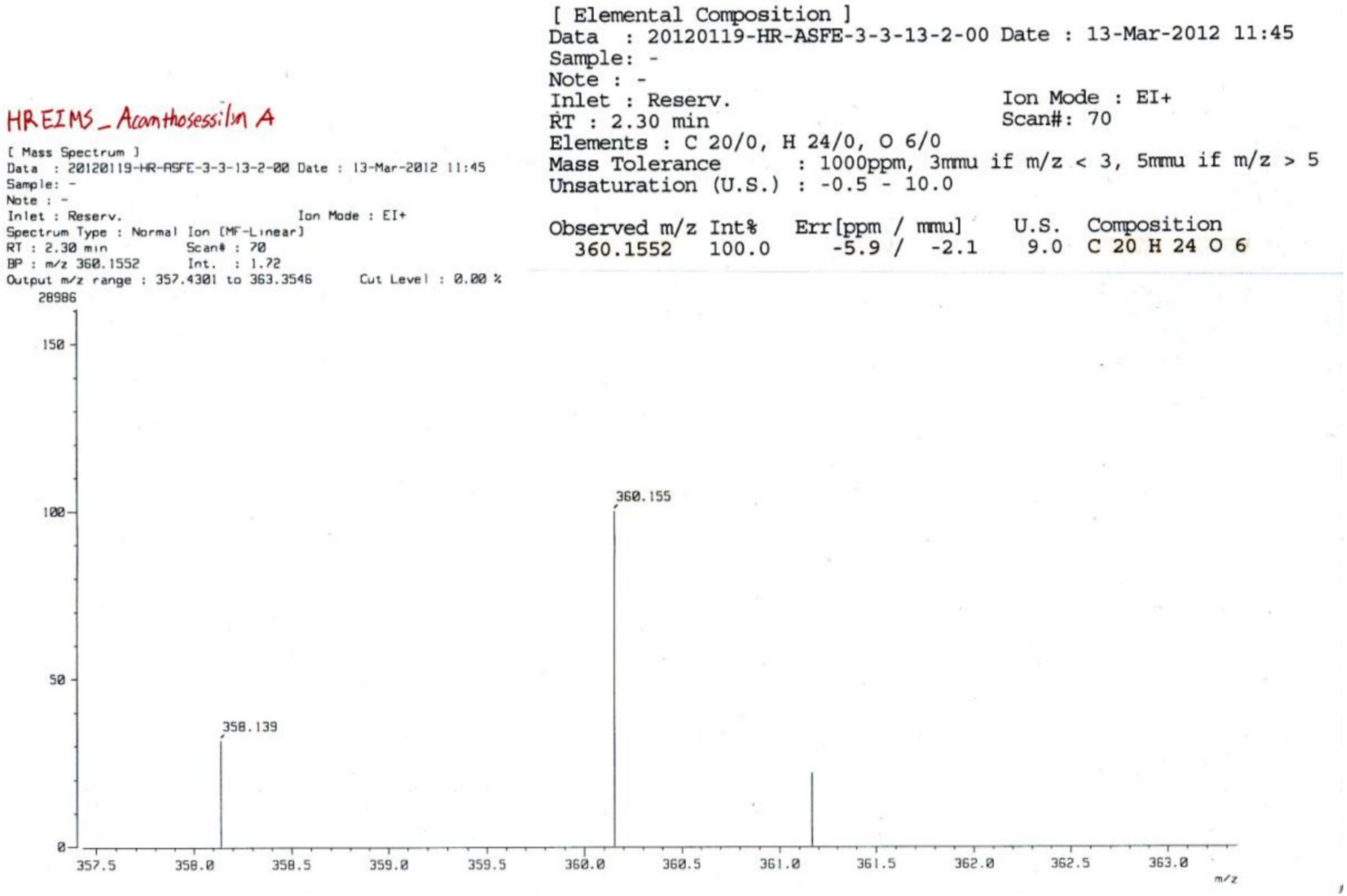

Supplement: Supplementary file 1 [file molecules-18-00041-s001.pdf]
